# Pyloroduodenal Duplication Cyst: The Rarest Alimentary Tract Duplication

**Published:** 2012-09-01

**Authors:** Bilal Mirza

**Affiliations:** Department of Pediatric Surgery, The Children's Hospital and the Institute of Child Health Lahore, Pakistan

**Keywords:** Pyloroduodenal duplication, Vomiting, Abdominal mass

## Abstract

Pyloroduodenal duplication is the rarest alimentary tract duplication known so far. A 1-year-old female patient presented with abdominal pain, lump in the epigastrium and occasional vomiting. On ultrasound and CT scan of abdomen suspicion of duplication cyst was made. Operation revealed a duplication cyst along pylorus and first part of the duodenum. Excision of free part and mucosal stripping of the attached part of the duplication was done.

## INTRODUCTION

Alimentary tract duplications commonly occur along ileum. Pyloroduodenal duplications are extremely rare variety of duplications with only 5 cases reported to best of our knowledge on literature search. These duplications present with abdominal mass and vomiting. In neonates, pyloroduodenal duplication can simulate pyloric stenosis [1-4]. A case of pyloroduodenal duplication is being reported. 

## CASE REPORT

A 1-year-old female was admitted with abdominal pain, abdominal lump and infrequent episodes of vomiting. The patient was born to a multi-gravida with no perinatal problem. The patient had infrequent episodes of non-bilious vomiting since birth for which occasionally medical treatment was sought. However, for the last 3 months the parents had noted an epigastric lump associated with abdominal pain. On examination, a vague mass was palpable in the epigastrium with no visceromegaly. There was tenderness on deep palpation. Ultrasound of the abdomen showed a 5 cm x 4 cm sized thick walled cyst in the epigastrium. CT scan of the abdomen also revealed a 5 cm x 5 cm sized cyst between the duodenum and the liver with wall enhancement, suggestive of duplication cyst (Fig. 1).

**Figure:**
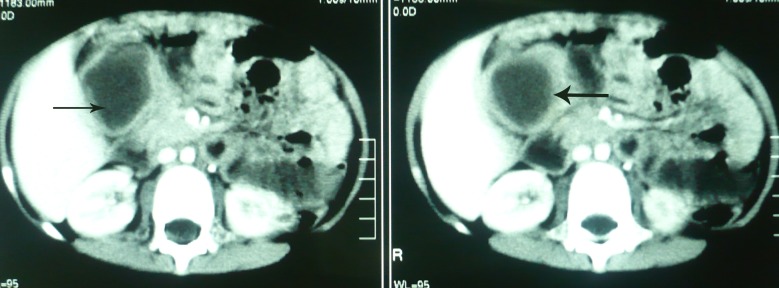
Figure 1: CT scan of the abdomen showing a hypo-dense cyst with wall enhancement.

Laboratory parameters were within normal limits. At operation, a cyst along the pylorus and first part of the duodenum was found (Fig. 2). The cyst was opened that showed a mucosal lined cavity having mucus in it (Fig. 3). As the cyst was not separable from the gut therefore, excision of the free part of the cyst and mucosal stripping of the rest was performed. The patient recovered well postoperatively. She is doing well at one year follow-up. The histopathology of the specimen showed a gastric mucosal lined tissue having smooth muscles in the wall.

**Figure:**
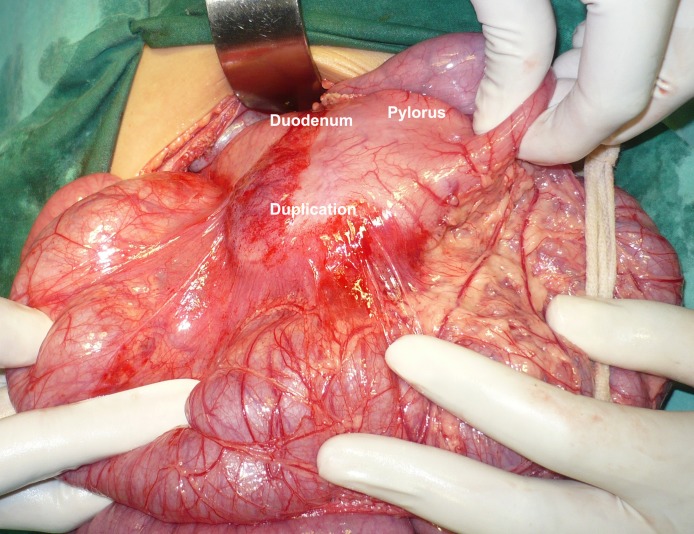
Figure 2: Duplication cyst along pylorus and duodenum.

**Figure:**
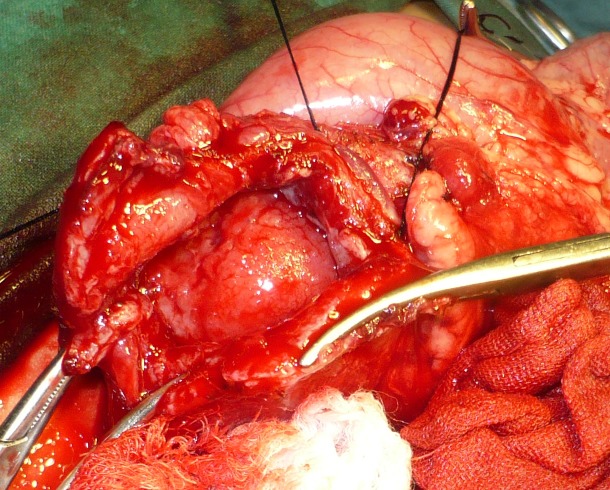
Figure 3: Cyst showing mucosal lining.

## DISCUSSION

Alimentary tract duplications are rare congenital anomalies. The duplications are named after the part of alimentary tract, to which these are intimately attached. Few cases of alimentary tract duplications may contain ectopic mucosa and pancreatic tissue [4]. Most of the alimentary tract duplications are cystic and non-communicating. Pyloroduodenal duplications are mostly non-communicating [1,2] as in our case; however a case of communicating pyloroduodenal duplication has also been reported [3]. 

Pyloroduodenal duplications present with symptoms of gastric outlet obstruction and abdominal mass. Similarly, our patient presented with abdominal mass and occasional emesis. The alimentary tract duplications, in this location, can mimic other cystic lesions like mesenteric cyst, pseudopancreatic cyst, and choledochal cyst etc. Plain abdominal radiograph may show a mass effect in the epigastrium surrounded by displaced gas shadows. Ultrasound, contrast study, endoscopy, and CT scan are important diagnostic tools. Thick wall of the cyst as appreciated on the CT scan, can give a clue of duplication cyst as wall enhancement is not featured by the most of other cystic lesions in the vicinity [1-3].

The Pyloroduodenal duplication has been managed by complete excision of the duplication with pyloroduodenal enterectomy and end to end pyloroduodenal anastomosis [2], or by excision of the free part of the duplication cyst along with mucosal stripping of the remaining portion which is intimately attached to the pyloro-duodenum [1]. The second approach was considered safe and was used in the index to which she responded well.

## Footnotes

**Source of Support:** Nil

**Conflict of Interest:** None declared
